# Cryptogenic multifocal ulcerative stenosing enteritis (CMUSE): a rare case and a review of the literature

**DOI:** 10.1093/jscr/rjaf977

**Published:** 2025-12-28

**Authors:** Lauren Hudson, Niamh Grayson, Desmond Toomey

**Affiliations:** Surgical Intern, University College Dublin, Midlands Regional Hospital Mullingar, Longford Rd, Robinstown (Levinge), Mullingar, Co. Westmeath, N91 W237, Ireland; Surgical Registrar, Royal College of Surgeons Ireland, Midlands Regional Hospital Mullingar, Longford Rd, Robinstown (Levinge), Mullingar, Co. Westmeath, N91 W237, Ireland; Consultant in General and Colorectal Surgery, Royal College of Surgeons Ireland, Midlands Regional Hospital Mullingar, Longford Rd, Robinstown (Levinge), Mullingar, Co. Westmeath, N91 W237, Ireland

**Keywords:** cryptogenic multifocal ulcerative stenosing enteritis, CMUSE, small intestine enteropathy, small intestine, enteropathy

## Abstract

Cryptogenic multifocal ulcerative stenosing enteritis (CMUSE) is a rare enteropathy of unknown aetiology and pathophysiology. It is characterized by chronic and recurrent ulceration and stenosis of the small intestine. We present the case of a 33 year old female who had multiple presentations to our hospital with symptoms of small bowel obstruction. Diagnostic laparoscopy revealed extensive serosal adhesions with mixed transmural inflammation. The mucosa was extensively ulcerated and the bowel wall was congested and haemorrhagic, with hypertrophy of the muscularis propria. Ileoscopy revealed multiple strictures. Following treatment with balloon dilatation and surgical resection, she responded to treatment with immunomodulators and has remained in remission. CMUSE is a rare but important differential of benign SI strictures. The mainstays of treatment are steroids, immunomodulators, balloon dilatations, and surgical resection. Treatment should be aimed at reducing steroid dependence and preserving small bowel length, resorting to surgical resection in cases of occluding strictures.

## Introduction

Cryptogenic multifocal ulcerative stenosing enteritis (CMUSE) is a rare enteropathy of unknown aetiology and pathophysiology. It is characterized by chronic and recurrent ulceration and stenosis of the small intestine (SI). These idiopathic multifocal strictures and ulcerations affect the SI exclusively [1]. Due to certain overlapping clinical and radiological features of more common aetiologies such as Crohn’s and non-steroidal anti-inflammatory drug (NSAID)-induced enteropathy, the diagnosis can be challenging [2]. We report a case of CMUSE in our institution and a review of the literature.

## Case presentation

A 33 year old female had multiple presentations to the emergency department with abdominal pain, nausea, and vomiting. Her past medical history included idiopathic intracranial hypertension; for which a VP shunt was inserted, and blindness in her left eye. She was a smoker with a 15 pack-year history. She categorically denied NSAID use and had no family history of inflammatory bowel disease (IBD).

An OGD was performed to investigate anaemia which showed reflux oesophagitis. She underwent colonoscopy to the terminal ileum, which was essentially normal. There was no evidence of IBD.

**Figure 1:**
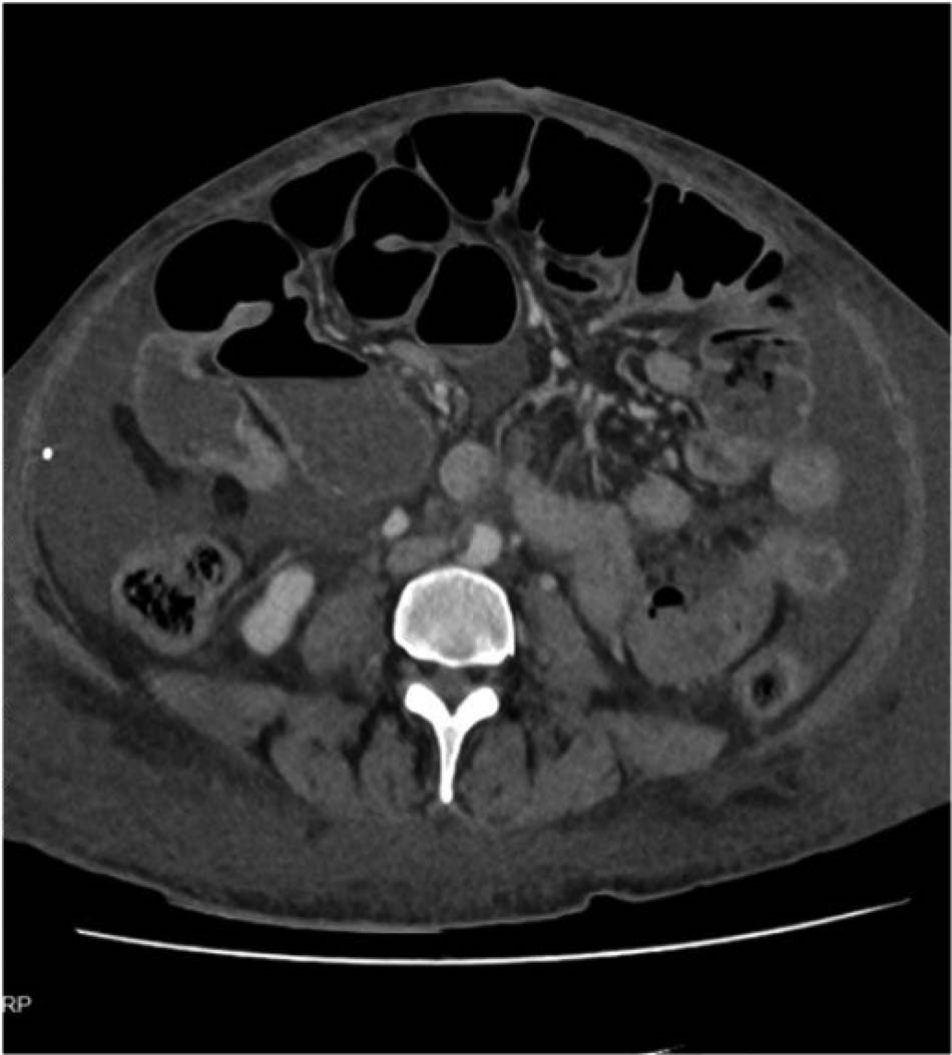
CT abdomen pelvis.

Three months later, she was admitted with anaemia(Hb:6 g/dL), profound hypoalbuminemia (albumin:15 g/L) and generalized oedema. Computed tomography (CT) showed dilated SI loops with a possible transition point in the right mid-abdomen in keeping with a multifocal mechanical bowel obstruction (Fig. 1).

She developed an acute small bowel obstruction (SBO) as an inpatient and a diagnostic laparoscopy was performed. This showed greater than 12 skip lesions in the distal ileum. The most proximal stricture causing obstruction was resected and a double barrel ileostomy was created.

The histology of the SI showed extensive serosal adhesions without transmural inflammation. The mucosa was extensively ulcerated and the bowel wall was congested and haemorrhagic, with hypertrophy of the muscularis propria. There was one section of withering of crypts with mucosal ulceration but no granulomata. It was reported as showing features of ischaemic enteritis with prominent ulceration and serositis. There were no features of Crohn’s disease.

Follow up ileoscopy showed an impassable inflammatory appearing stricture at 10 cm of the efferent limb. There were multiple areas of stricture formation in the afferent limb; not causing obstruction. Biopsies were taken from both areas. A vasculitic and igG4 screen were sent at this time which were both negative.

She was referred for an anterograde double balloon enteroscopy which showed ileal disease with ileitis, stenosis, and an ulcer. The histology was reviewed at an MDT meeting and a working diagnosis of CMUSE was made. She was commenced on Adalimumab. She continued to smoke following diagnosis.

**Figure 2:**
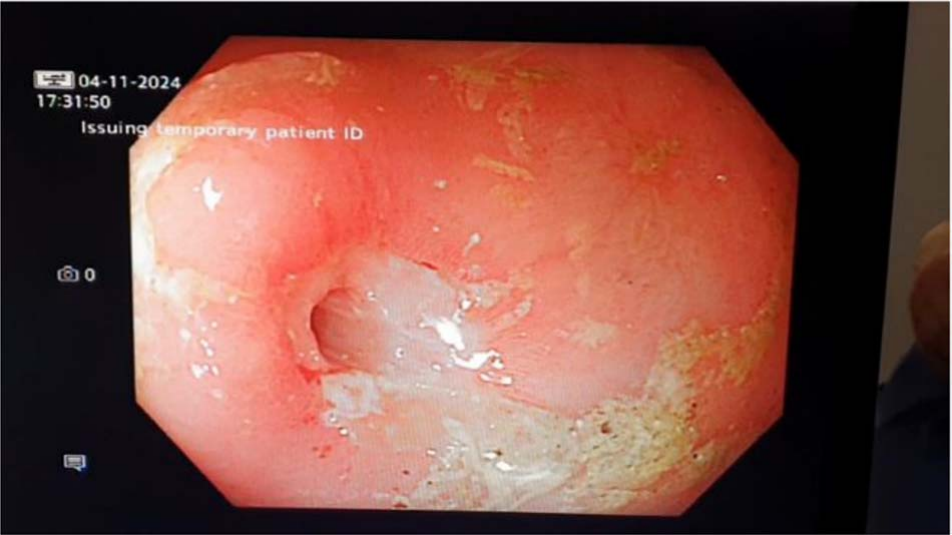
Small bowel stricture at endoscopy.

Eight months later she presented to the emergency department with symptoms of SBO. A balloon dilatation of 3 strictures in the obstructing segment was performed (Fig. 2).

**Figure 3:**
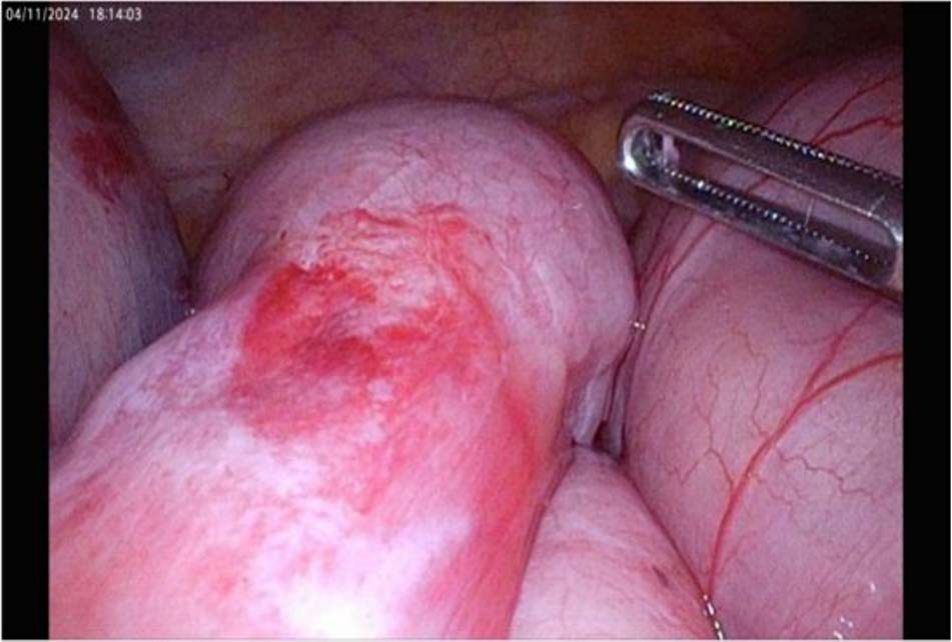
Inflammation evident at laparoscopy.

Her symptoms settled initially but she later re-presented with SBO. At this point a laparoscopic resection of the obstructing segment of small bowel, and a refashioning of her stoma was undertaken (Fig. 3). 20 cm of ileum was resected with three tight strictures noted.

She recovered well post-operatively and was seen back in the Gastroenterology clinic. She was recommenced on Adalimumab and commenced on Azathioprine. She has had no further presentations in 9 months.

## Discussion

### CMUSE

CMUSE was first described in 1964 by Debray *et al*. [3]. It is characterized by chronic and recurrent stenosis from idiopathic multifocal strictures and ulcerations of the SI exclusively. Findings include short, superficial, multifocal small bowel strictures <2 cm with circumferential luminal narrowing and stratified hyperenchancement [3].

### Symptoms

Characteristic symptoms of CMUSE include abdominal cramping, gastrointestinal bleeding, and anaemia, with a relapsing–remitting course despite surgery and immunosuppressive treatment [2]. Lower limb swelling secondary to hypoalbuminenia and anaemic symptoms are also reported [1].

### Pathophysiology

The aetiology and pathogenesis of CMUSE remains unknown. Some experts speculate that autoimmune abnormalities, excessive formation of fibrous tissue, vasculitis, and mutations of PLA2G4A contributory to its pathogenesis [4–6].

Chronic nonspecific multiple ulcers of the small intestine is a rare autosomal recessive inherited enteropathy characterized by persistent blood and protein loss from the small intestine; similar to the presentation of CMUSE. Some studies have found that SLCO2A1 mutations exist in some patients with ulcerative small-intestinal strictures, so it is also known as chronic enteropathy associated with the SLCO2A1 gene (CEAS) [7]. The mutant SLCO2A1 protein for each mutation exhibited impaired prostaglandin transport. This description generally appears in Japan, and the link between CEAS and CMUSE has not yet been clarified.

### Diagnosis

The diagnosis of CMUSE can be challenging, due to overlapping clinical and radiological features with other diseases [8]. They may be treated for other more common aetiologies, such as Crohn’s disease, and may undergo multiple surgical resections due to recurrent strictures.

Table 1 below summarizes features that are helpful in differentiating CMUSE from other enteropathies.

**Table 1:** Differentiating features between CMUSE, Crohn’s disease, NSAID-induced enteropathy, and intestinal tuberculosis

Features	CMUSE	Crohn’s disease	NSAID-induced enteropathy	Intestinal tuberculosis
Clinical	Absence of the typical extraintestinal manifestations such as cutaneous erythema nodosum, arthritis, and eye disease. Lack of NSAID use in the patient’s history and no gradual recovery after discontinuation. Lack of exposure to Tuberculosis, negative Quantiferon testing, and a lack of Tuberculosis lesions on chest CT	Presence of typical extraintestinal manifestations such as cutaneous erythema nodosum, arthritis, and eye disease.	History of NSAID use. Gradual recovery after discontinuation.	History of Tuberculosis exposure, positive Quantiferon testing. Evidence of Tuberculosis lesions on CT. T-SPOT.TB is a new-generation immunological diagnostic technique for detection of tuberculosis infection. The immune response caused by Tuberculosis antigens can be measured by the above tests to determine whether there is *Mycobacterium tuberculosis* infection [8].
Endoscopic	Multifocal, pleiomorphous ulcers, not reaching the mucosal proper layer, which are sharply demarcated from the normal mucosa. The ulcerations are limited to the SI and there is a lack of systemic inflammation and fistula/fissure formation.	Skip lesions and cobblestone appearance, often in an ileocolic distribution [2]. There may be fistula and fissure formation.	Multiple stricture sites with semi-circular ulcers (usually with active haemorrhage) or annular constrictions of the mucosa and submucosa leading to an obstructed lumen [2]. Unlike CMUSE, NSAID-induced injury also occurs in the stomach and the colon.	Lesions are found mostly in the ileocecal junction and terminal ileum, and the lesions show annular ulcers. Caseating granulomas and *M. tuberculosis* can be detected by enteroscopic biopsy [8].
Histology	Non-specific inflammation, erosion and submucosal fibrosis. There may be vascular changes without transmural inflammation [2].	Transmural inflammation with widening of the submucosa by oedema and inflammatory infiltrate, scattered aggregations of granulomatous lymphoid tissue [2].	Ulceration (including diffuse loss of villi, mucosal and submucosal neutrophilic exudates) and transmural inflammation [2].	

Capsule endoscopy and double balloon enteroscopy are typically used to investigate CMUSE. As the SI is a long and tortuous segment of the digestive tract, the newly released motorized spiral enteroscope may allow deeper enteroscopy as compared to the other available models of device-assisted enteroscopy [9].

Genetic analysis is helpful in the diagnosis of CMUSE, and future studies may clarify the relationship between CMUSE and CEAS, and the role of screening for SLCO2A1 and PLA2G4A gene mutations.

### Management

CMUSE is steroid-sensitive in majority of cases, however up to 50%–66% of patients become steroid dependent. 20 mg of prednisolone daily can reduce risk of steroid dependency while causing regression of disease [4].

Immunomodulators are an important steroid-sparing treatment [9]. Upadacitinib is a selective Janus kinase 1 inhibitor. It may help regulate aberrant immune activation in CMUSE, potentially reducing inflammation, and mitigating ulcer formation and stricture development.

Balloon enteroscopy enables accurate diagnosis and treatment of tight stenoses, potentially reducing need for surgical resection [4].

Patients often undergo multiple surgical resections of obstructing lesions prior to diagnosis of CMUSE. Treatment should be aimed at preserving small bowel length, with utilization of surgical resection for occluding strictures or steroid-refractory disease.

### Prognosis

As seen with our patient, treatment with immunomodulators such as Azathioprine, Adalimumab, and Upadacitinib can help to maintain remission and prevent further hospital admissions and bowel resections. Low dose steroids also have a role in maintaining remission. Patients may still require dilatations with double balloon enteroscopy or surgical resections for occluding strictures.

While management for both CMUSE and other conditions such as Crohn’s overlap, accurate differentiation is essential. In this case, multiple biopsies revealed no features of Crohn’s disease, so treatment would not have been initiated. Confirming CMUSE allowed a comprehensive management plan to be developed; including surgery, endoscopic dilatations, and ongoing medical therapy.

## Learning points/take home messages

CMUSE is a rare but important differential of benign SI strictures.

Due to overlapping clinical and radiological features of more common aetiologies such as Crohn’s and NSAID-induced enteropathy, the diagnosis can be challenging.

A multidisciplinary approach needed to confirm diagnosis.

The gold standard investigations for this condition are capsule endoscopy or double balloon enteroscopy, with histology confirming diagnosis.

The mainstays of treatment are steroids, immunomodulators, balloon dilatations, and surgical resection.

Treatment should be aimed at reducing steroid dependence and preserving small bowel length, resorting to surgical resection in cases of occluding strictures.
